# Survival of influenza virus H1N1 and murine norovirus in raw milk cheeses

**DOI:** 10.1128/aem.01881-25

**Published:** 2025-10-31

**Authors:** Madeleine Blondin-Brosseau, Wanyue Zhang, Caroline Gravel, Jennifer Harlow, Xuguang Li, Neda Nasheri

**Affiliations:** 1 Bureau Microbial Hazards, Health Canada 6348, Ottawa, Ontario, Canada; 2 Centre for Oncology, Radiopharmaceuticals and Regulatory Research, Health Canada 6348, Ottawa, Ontario, Canada; 3 Department of Biochemistry, Microbiology and Immunology, University of Ottawa 6363, Ottawa, Ontario, Canada; Universita degli Studi di Napoli Federico II, Portici, Italy

**Keywords:** influenza virus H1N1, murine norovirus, raw milk cheese, aging, survival

## Abstract

The spillover of highly pathogenic avian influenza (HPAI) to dairy cattle and its presence at high titers in raw milk has created a concern that consumption of dairy products made from unpasteurized milk could result in human exposure. We examined whether H1N1, as a surrogate for HPAI viruses, and murine norovirus (MNV), as a surrogate for unenveloped enteric viruses, can survive the cheese-making process by making cream cheese-style and feta-style cheeses from artificially inoculated raw milk. We used the modified ISO 15216 method for viral extraction, which has a limit of detection of 3.0–3.7 log for infectious H1N1 in soft cheeses. Infectious H1N1 virus was detected in the curd from both types of cheeses and in the whey from cream cheese but not in the whey from feta cheese. Infectious MNV particles were detected in the curd and in the whey for both types of cheeses. To study the effect of salt, pH, aging, and microbiome of various unpasteurized milk cheeses on viral survival, we artificially inoculated different commercial unpasteurized milk cheeses with 7 log PFU of H1N1 and 6 log PFU of MNV and examined viral survival over an 8 week period at 4°C. We observed that H1N1 survived for 8 weeks, with an average of 2.6, 2.75, and 4.0 log reductions on cheddar, washed rind firm cheese, and semisoft brie-like cheese, respectively (*P* < 0.001). The level of infectious H1N1 fell below the limit of detection in cream cheese after 2 weeks of inoculation. It is noteworthy that the sensory qualities of soft and semisoft cheeses deteriorated in 2 weeks, but the sensory qualities of firm cheeses did not change drastically, demonstrating that, as expected, they endure the aging process better than the soft and semisoft cheeses. Furthermore, the limit of detection for soft cheeses is approximately 1 log lower compared to firm cheeses, and the extraction method has a lower efficiency for soft cheeses compared to firm cheeses. MNV survived in firm cheeses for 8 weeks with only 1 log reduction on cheddar cheese and 2 log reductions on the washed rind firm cheese (*P* < 0.05). The data obtained in this study could help with the risk assessment of dairy products made from unpasteurized milk.

**IMPORTANCE:**

The rapid spread of H5N1 viruses among dairy cattle in the United States and the viral shedding at high titers raised concern regarding the safety of dairy products, specifically cheeses made with unpasteurized milk. In this study, we demonstrated that murine norovirus and influenza virus can survive the cheese-making and aging process. Therefore, it is recommended that milk contaminated with viruses be heat-treated to ensure safety.

## INTRODUCTION

Since their emergence in 1996, highly pathogenic avian influenza (HPAI) H5N1 viruses have posed a persistent threat to human and animal health, with significant morbidity and mortality in wild birds, domestic poultry, and mammals (1). As of December 2021, clade 2.3.4.4b HPAI H5N1 viruses have spread around the world and become the predominant subtype (2), while unlike previous intercontinental waves, these viruses continue to circulate widely among wild birds and cause panzootic transmissions. The unusual transmission of clade 2.3.4.4b H5N1 viruses to goats and dairy cattle has raised concerns about the risk of foodborne exposure to humans through consumption of dairy products and the possibility of outbreaks of severe illnesses. To date, it is unclear whether consumption of dairy products contaminated with H5N1 would result in infection in humans; however, multiple reports indicated that drinking contaminated raw milk led to severe infection in mice and cats (3, 4), and orogastric inoculation of cynomolgus macaques caused a mild infection (5).

While it has been demonstrated that H5N1 RNA can be detected and quantified in cheeses made with pasteurized, naturally contaminated milk, no infectious virus was detected (6). This finding further confirms that pasteurization is effective in inactivation of influenza viruses (7, 8). However, the fate of influenza viruses in cheeses made from unpasteurized milk is not well studied. In Canada, selling unpasteurized milk cheeses is permitted if they meet certain conditions, such as storage for at least 60 days (9). Although certain jurisdictions, such as Quebec, have regulations that can allow cheese not aged for a minimum of 60 days under specific conditions (10).

Historically, one of the earliest foodborne viral outbreaks was attributed to milk contaminated with the poliovirus (11). Since then, outbreaks of tick-borne encephalitis virus (TBEV) and hepatitis A virus have also been associated with dairy products (12, 13). Nevertheless, dairy products are not considered common vehicles for transmission of foodborne viruses, and so, little is known about viral survival during cheese making and curing/aging.

The aim of this study was to assess the survival of influenza viruses and typical foodborne viruses during the process of unpasteurized milk cheese making and aging. Since working with HPAI viruses, including H5N1, requires biosafety level (BSL)-3 facilities, we chose H1N1, which is a BSL-2 agent, as a surrogate for H5N1 viruses. Murine norovirus (MNV) was employed as a surrogate for common unenveloped foodborne viruses such as human norovirus. We first examined whether H1N1 and MNV endure the cheese-making process by making raw milk cream cheese-style cheeses and feta-style cheeses in the lab from artificially inoculated raw milk. Next, we analyzed viral stability during the aging process by spiking commercially available unpasteurized milk cheeses and testing for the presence of infectious virus at 4°C over an 8 week period.

## MATERIALS AND METHODS

### Cells and viruses

Madin-Darby canine kidney (MDCK) cells were grown in Dulbecco’s modified Eagle medium (DMEM) supplemented with 10% heat-inactivated fetal bovine serum, 25 mM HEPES, 1.5 g/L sodium bicarbonate, 20 U/mL penicillin, and 0.02 mg/mL streptomycin. The A/Netherlands/602/09 (H1N1) virus was a gift from Dr. Florian Krammer (Icahn School of Medicine at Mount Sinai). The virus was amplified in 10-day-old embryonated chicken eggs for 2 days at 37°C. After cooling overnight at 4°C, the allantoic fluid was collected and centrifuged. The supernatant was then filtered through a 0.45 µm filter and sucrose-purified. The virus titer was determined by plaque assay on MDCK cells. The purified virus was used for spiking the milk and cheese samples.

For MNV stock preparation, murine BV-2 cells were inoculated with MNV-1 (kindly provided by Dr. Virgin, Washington University School of Medicine, St. Louis, MO, USA). The virus was then extracted after approximately 48 h incubation by three cycles of freeze (at −80°C) and thaw (at room temperature) as described previously (14).

### H1N1 plaque assay

Dilutions of the cheese or milk extracts were prepared in serum-free DMEM supplemented with 0.2% bovine serum albumin (BSA), 25 mM HEPES, and 1.5 g/L sodium bicarbonate. Confluent MDCK monolayers in six-well plates were then rinsed and incubated with the homogenates at 37°C for 2 h. After removing the inoculum, the cells were rinsed and overlaid with DMEM containing 0.2% BSA, 25 mM HEPES, 1.5 g/L sodium bicarbonate, 40 U/mL penicillin, 0.04 mg/mL streptomycin, 2 μg/mL tosyl phenylalanyl chloromethyl ketone (TPCK)-treated trypsin, and 0.8% agarose. Cells were incubated at 37°C and 5% CO_2_. After 3 days, the cells were fixed using 3.7% paraformaldehyde for 4 h, then the overlay was removed, and the cells were stained with crystal violet for plaque counting.

### MNV plaque assay

For MNV, plaque assay was performed in BV-2 cells in a 12-well plate using the procedures that have been described previously (15).

### Raw milk

The cow raw milk used to make the homemade cheeses was obtained from the Canada Agriculture and Food Museum, Ottawa, ON, in June 2024, July 2024, and August 2025. The milk contained approximately 4.3% fat, 3.18% protein, and 5.85% lactose.

### Artificial inoculation of raw milk

Two hundred milliliters of raw milk was artificially inoculated with 10 µL of 1.9 × 10^9^ PFU of H1N1 viruses and 40 µL of 1.0 × 10^9^ PFU of MNV in three biological replicates. This created an initial concentration of H1N1 of 9.5 × 10^6^ PFU/mL and 5.0 × 10^6^ PFU/mL of MNV.

### Preparation of homemade cream cheese-style cheese

The Swiss-style cream cheese was prepared using the recipe and ingredients of a commercial kit for homemade cheese (Soft Cheese Kit, Cat. 31104; Glengarry Cheese Inc, Lancaster, ON). The kit provided a curd draining bag, stainless steel dairy thermometer, basket cheese molds, cheese bandage netting, bacterial cultures (mesophilic Aroma B, MT1, and MA 4001 5dcu), rennet, and calcium chloride. The original recipe for Swiss cream cheese called for a much larger volume of milk; therefore, all quantities were calculated for 200 mL of raw milk. The spiked raw milk, 200 mL, was warmed to 30°C while mixing. To start the cheese-making process, 0.0615 g of mesophilic Aroma B culture mix and 1 drop of rennet were added to the milk. The beaker was covered and placed in a biosafety cabinet to allow the milk to set at room temperature until a firm curd formed (approximately 16 h). Once the curd and the whey were clearly separated, both components were poured into a cheesecloth and held in place by two superposed colanders to drip for 8 h, over a second beaker to collect all the whey. Once drained, the cheese and the whey were transferred into separate airtight containers and stored at 2°C–8°C until testing. A final quantity of 47 g of cheese and 145 g of whey was obtained. For testing by plaque assay, 2.5 g of cheese was transferred into a falcon tube, in triplicate, and contents of each tube were subjected to extraction as described below. To test the whey, 1 mL aliquot was directly used without additional processing.

### Preparation of the mesophilic starter culture (used for the homemade feta-style cheese)

The ingredients and method provided in the Soft Cheese Kit (Cat. 31104) from Glengarry Cheese Inc. were used as a reference. To ensure the starter would only contain the expected culture, 1 L of commercially pasteurized 3.25% milk was transferred to a lidded glass bottle and autoclaved. The milk was cooled to room temperature, and 3/8 teaspoon (1.8 mL) of the freeze-dried mesophilic Aroma B starter was added to the sterile milk. The powder was allowed to dissolve for 2 minutes and then thoroughly mixed in before placing the jar at room temperature for 16 h (until the milk thickened). The starter culture (pH 4) was then stored at 2°C–8°C for up to a month.

### Preparation of feta-style cheese

The ingredients and method provided in the Soft Cheese Kit (Cat. 31104) from Glengarry Cheese Inc. were used to make the feta-style cheese. The spiked raw milk, 200 mL, was warmed up to 30°C in a large beaker on a heating block while mixing. To start the cheese-making process, 4.2 mL of prepared mesophilic starter culture (pH 4) was added and thoroughly mixed using a top-and-bottom stir technique. The milk was set for 45 minutes while maintaining the temperature at 30°C. Lipase powder (0.025 g) and rennet (64 µL) were mixed in before allowing the mix to set to form a firm curd for 30 minutes at 30°C. The curds were then cut into cubes using a disposable spatula and allowed to rest for 5 minutes. After that time, the curds were gently stirred for 20 minutes while still maintaining the temperature at 30°C. The curds were then allowed to settle, and the majority of the separated whey was transferred to an airtight container using a disposable serological pipette. The rest of the mixture was poured into a cheesecloth draining bag held in place by two superposed colanders and let to drip for 8 h, over a second beaker to collect all the whey. Once drained, the curd and the remaining whey were transferred into separate airtight containers and stored at 2°C–8°C until testing. A final quantity of 40 g of cheese and 143 g of whey was obtained. For testing by plaque assay, 2.5 g of cheese was transferred into a falcon tube, in triplicate, right after the preparation (*T* = 0 h). To test the whey, 1 mL aliquot was directly used without additional processing.

### Commercial unpasteurized milk cheese samples

Four types of commercial unpasteurized milk cheeses were purchased from local grocery stores in the National Capital Region in ON and QC, Canada: soft cheese (not aged), mixed rind semisoft brie-like cheese, cheddar, and a washed rind firm aged cheese. These cheeses contain approximately 12%, 27%, 31% and 38% of fat, respectively (Table 1), and their salt content ranged from 130 to 150 mg per 30 g (Table 1).

**TABLE 1:** Fat, moisture, and salt content per 30 g of the commercial unpasteurized milk cheese

Type of cheese	Commercial name	% Fat	% Moisture	Salt (mg)	pH
Washed rind firm cheese	Louis d'or	32–38	35	140	5.4
Firm cheese (cheddar)	Le cru du clocher	31	39	130	5.4
Mixed rind, semisoft cheese	Pied-de-Vent	27	49	150	5.5
Soft cheese	Péningouin	12	56	130	4.9

### Artificial inoculation of the commercial unpasteurized milk cheese samples

For each commercial cheese and each time point (weeks 0–8), 2.5 g of cheese was weighed out as triplicate samples. In total, 27 tubes were prepared per cheese. For the soft and semisoft cheeses, the samples were weighed directly into 50 mL falcon tubes. For the firm cheeses, the samples were weighed and chopped finely with a scalpel before being added to the 50 mL falcon tubes. Each sample was then spiked with 10 µL of the 9 log PFU/mL stock of H1N1 viral stock and 10 µL of 8 log PFU/mL of MNV viral stock using a micropipette to place drops on the cheese surface. All the samples were then stored in a VWR double glass-door refrigerator (model GDM-49), where the temperature fluctuated between 3.2°C and 4.4°C, until the appropriate time point.

### Virus extraction methods

For the extraction of viruses from the cheese samples, we used a modification of the ISO 15216 method for leafy greens (16). We have already demonstrated that this method is effective in the extraction of viruses from a variety of cheeses (17). Samples of cheese (2.5 g) were suspended in 4 mL of Tris glycine 1% beef extract buffer (50 mM glycine, 100 mM Tris, 1% [wt/vol] beef extract [pH 9.5]). The samples were then homogenized (Fisherbrand, Homogenizer 850), using a 10 mm probe until homogenous, for 20–60 seconds at a speed setting of 15,000 rpm. All samples were then centrifuged at 6,000 × *g* for 10 minutes at room temperature before decanting the supernatant by carefully pouring the liquid into a new 15 mL falcon tube. Samples were then stored on ice until testing by plaque assay.

### Determination of *D*-values

The inactivation kinetics of H1N1 in unpasteurized milk cheese were calculated based on the decimal reduction time (*D*-value). The *D*-value is defined as the time required at a specific temperature to achieve a 1 log reduction in viral titer, and it is calculated at 4°C from the negative reciprocal of the slopes of the regression lines; i.e., *D* = −1/slope, where the slope is derived from the plot of log_10_ of surviving viruses (PFU/2.5 g) versus aging time (days).

### Statistical analysis

Statistical analysis was performed using GraphPad Prism v.9.0 (GraphPad Software) and the unpaired *t*-test method.

## RESULTS

### Viral survival during cheese making

We inoculated raw milk with a total of 7.3 log PFU of H1N1 and 7.6 log PFU of MNV and proceeded with making feta-style cheese and cream cheese-style cheese in three independent experiments. The pH values from the cheeses and their components are shown in Table 2. The presence of infectious virus in the cheese curds and whey was assessed by plaque assay. As indicated in Table 3, infectious H1N1 was detected in the cream cheese curd in all three experiments and in the cream cheese whey from one experiment. On the other hand, infectious H1N1 was detected in the feta curd from two experiments, and no infectious H1N1 was detected in the whey from feta. It is noteworthy that viral suspension extracted from the feta cheese had cytotoxicity effect, and higher dilutions (1:20 compared to 1:2 and 1:5 for cream cheese) had to be tested for plaque assay. This could partly explain the lack of infectious viral detection for experiment #2 and low viral titer detection for experiment #3 (Table 3).

**TABLE 2:** pH from the cheeses and their components

Product	pH
Rennet	5.2
Starter	4.4
Raw milk	6.55
Cream cheese whey	5.1
Cream cheese	6.5
Feta whey	4.7
Feta cheese	6.5

**TABLE 3:** Detected infectious H1N1 in PFU in 2.5 g of curd and 1 mL whey of the lab-made cheeses ± standard deviation^*a*^

	Experiment #1	Experiment #2	Experiment #3
Cream cheese	32 ± 3	123 ± 53	35 ± 6
Cream cheese whey	ND	22 ± 6	ND
Feta	35 ± 2	ND	8 ± 1
Feta whey	ND	ND	ND

^*a*^ ND, not detected.

Given that we obtained approximately 47 g of cream cheese, the total number of detected H1N1 is about 600 (2.8 log) PFUs at the end of the production process. We expect that nearly 3–4 log PFUs present in the sample would be lost due to limitations in the virus extraction method and infectivity assay, as the recovery rate for detection of infectious H1N1 from soft cheeses is approximately 0.2% (17). Thus, we estimate that 1.5–0.5 log virus was inactivated during the cheese-making process. Altogether, these data demonstrate that the cheese-making process did not markedly inactivate H1N1.

Infectious MNV particles were detected in both cream cheese and feta curds and in their respective whey in all experiments (Table 4). The number of MNV infectious particles isolated from both cream cheese and feta cheese was approximately 1 log higher than H1N1, i.e., 325 and 567 PFUs per 2.5 g of cheese. However, we know that the recovery rate for extraction of infectious MNV is 25 times higher than for H1N1 (17), and therefore a combination of higher extraction efficiency and higher tolerance to the cheese-making process has led to the higher presence of MNV in the curd compared to H1N1. MNV was also detected in the whey from both cream cheese and feta cheese, although a higher titer of MNV was recovered in the whey from cream cheese compared to feta cheese (*P* < 0.01) (Table 4). The difference for viral presence between the curd and whey was statistically significant for both cream cheese and feta cheese, with *P* = 0.05 for cream cheese and *P* < 0.0001 for feta cheese.

**TABLE 4:** Detected infectious MNV in PFU in 2.5 g of curd and 1 mL whey of the lab-made cheeses^*a*^

	MNV
Cream cheese	295 ± 24
Cream cheese whey	192 ± 60
Feta	567 ± 32
Feta whey	20 ± 10

^*a*^ Data are the average of three experiments, ±standard deviation.

### Viral survival during cheese aging

**Fig 1:**
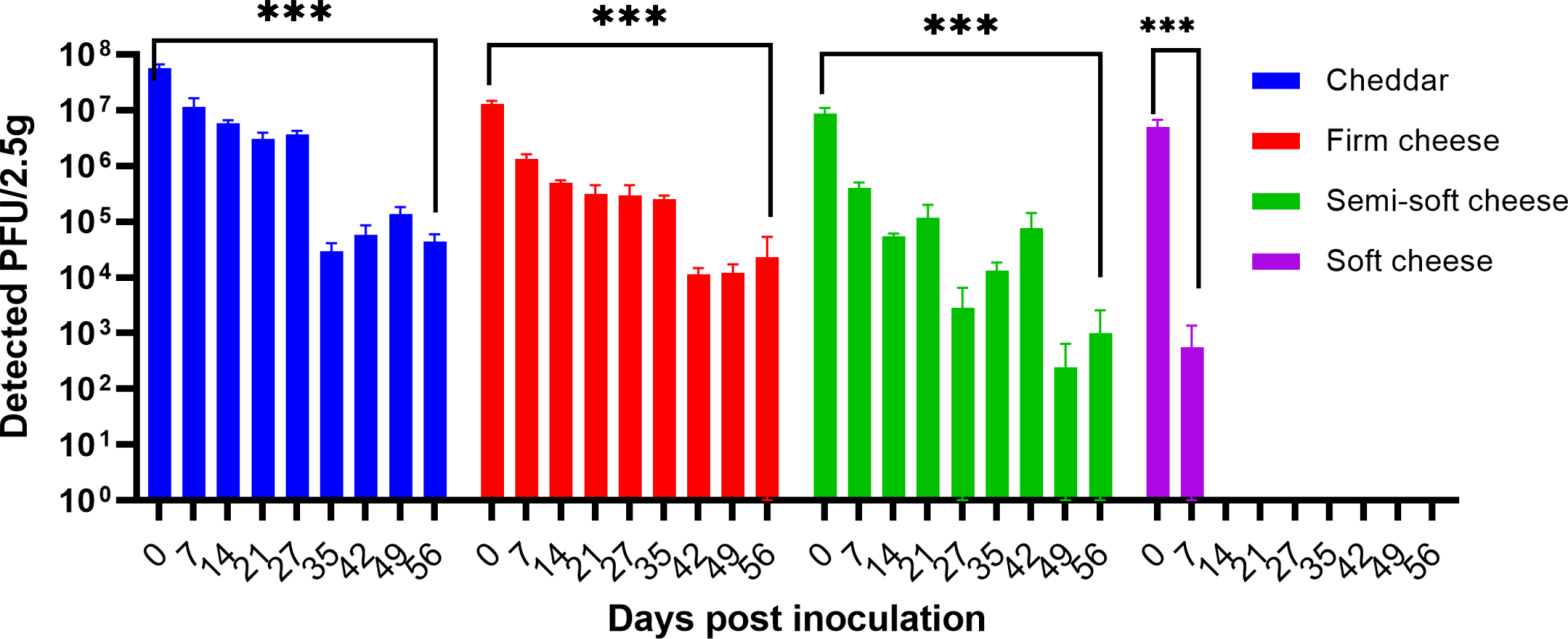
Survival of H1N1 during an 8 week aging period on cheddar, washed rind firm cheese, semisoft cheese, and soft cheese. The data are the average of three independent experiments, and error bars represent standard deviations. ***P < 0.001, calculated by *t*-test.

Next, we evaluated the stability of H1N1 and MNV during cheese aging or curing by artificially inoculating various commercial unpasteurized milk cheeses and storing them at 4°C over an 8 week period. Cheese samples were collected on days 0, 7, 14, 21, 28, 35, 42, 49, and 56. Viral infectivity was tested by plaque assay. As shown in Fig. 1, except for soft cheese, H1N1 survived for 8 weeks, with approximately 2.6 log, 2.75 log, and 4 log reduction on cheddar, washed rind firm cheese, and semisoft brie-like cheese, respectively. The reduction between day 0 and day 56 is statistically significant for all three cheeses. We did not detect any infectious H1N1 on soft cheese in week 2 onward. It is noteworthy that the sensory qualities of the soft and semisoft cheeses deteriorated markedly after 2 weeks. This included the appearance of mold and changes in the consistency and color of the soft and semisoft cheeses. Mold also formed on the firm cheeses from week 6 onward.

The decimal reduction times (*D*-value) were estimated for H1N1 on each cheese (Table 5). As expected, the *D*-values for H1N1 on firm cheeses were greater than those for the soft and semisoft cheeses: 20.5 ± 3.4 days and 17.3 ± 3.2 days for cheddar and washed rind firm cheese, respectively, versus 16.1 ± 3.8 days and 1.8 ± 0.1 days for semisoft cheese and soft cheese, respectively. The statistically significant difference is only observed compared to cream cheese (*P* < 0.01).

**TABLE 5:** Determination of decimal reduction times (*D*-values) in days ± standard deviation^*a*^

	H1N1	MNV
Cheddar	20.5 ± 3.4	63.7 ± 12.5
Firm cheese	17.3 ± 3.2	26.4 ± 4.0
Brie	16.1 ± 3.8	ND
Cream cheese	1.8 ± 0.1	ND

^*a*^ ND, not determined.

**Fig 2:**
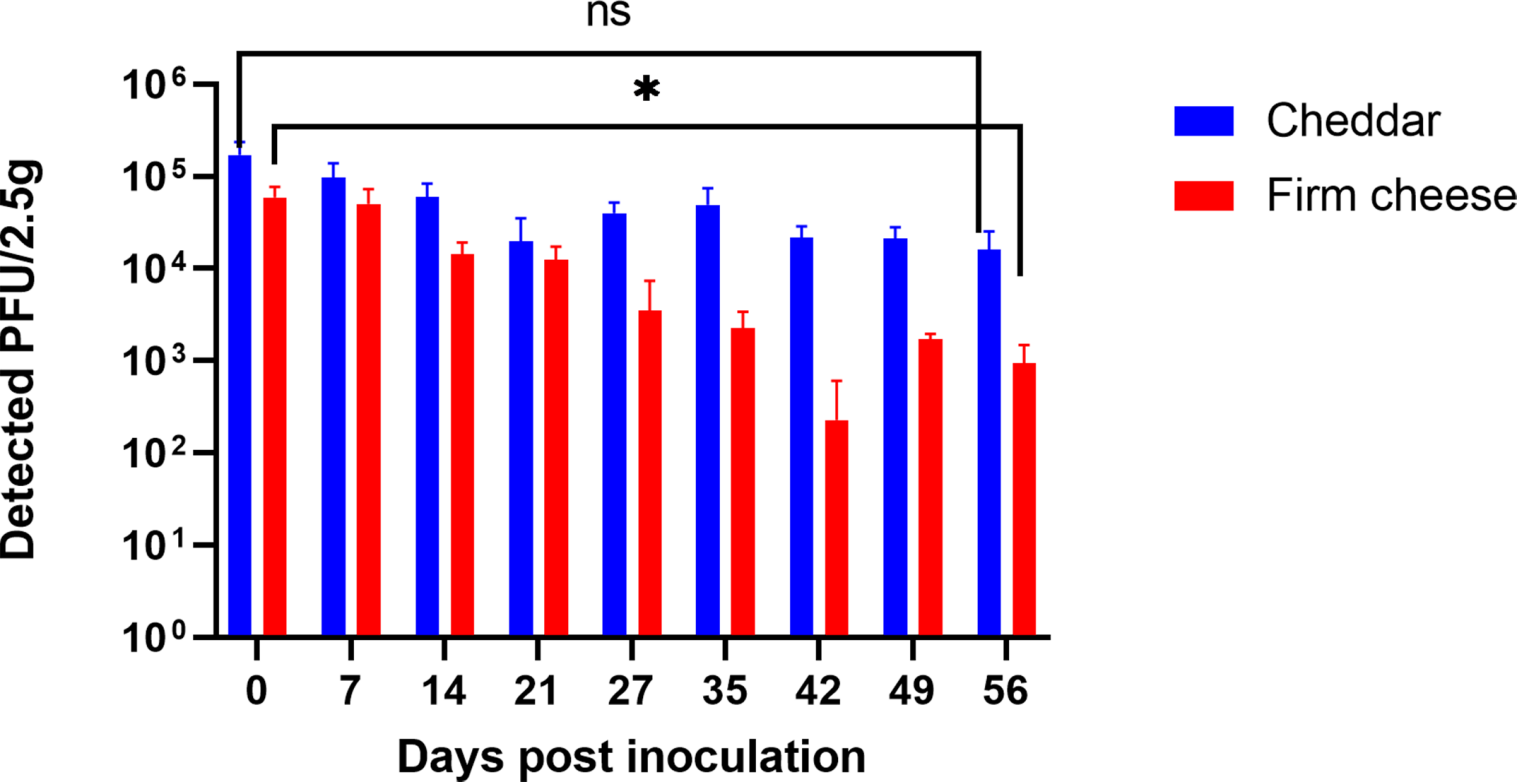
Survival of MNV during 8 weeks of aging on a washed rind firm cheese and cheddar cheese. The data are the average of three independent experiments, and error bars represent standard deviations. ns, non-specific. *P < 0.05 calculated by *t*-test, ns is not significant.

We also evaluated the survival of MNV in firm cheeses over an 8 week period, and we saw approximately 1 log reduction on cheddar, which is not statistically significant, and 1.7 log reduction on the washed rind firm cheese (Fig. 2). The *D*-value for MNV on cheddar was higher (63.7 ± 12.5 days) compared to the washed rind firm cheese (26.4 ± 4.0 days) (*P* < 0.01) (Table 3).

## DISCUSSION

Although several studies have shown that pasteurization is effective against inactivation of H5N1, consumption of raw milk and raw milk cheeses could represent an exposure risk to infectious virus. Currently, there is evidence that the consumption of raw milk cheeses made with milk contaminated with TBEV could result in human infection (18), demonstrating that the process of cheese making is not fully effective in eliminating this enveloped virus. Thus, we set out to study the impact of the process of cheese making and aging on the survival of human influenza virus H1N1, as a surrogate for avian influenza viruses, and MNV, as a surrogate for unenveloped foodborne viruses such as norovirus. We did detect infectious H1N1 in the cream cheese curd from all three experiments and infectious H1N1 in the feta cheese curd of two out of three experiments. It is noteworthy that the viral extract from feta cheese demonstrated cytotoxicity and caused damage to the MDCK monolayer. Therefore, higher dilutions (1:20 or higher) of viral suspension had to be tested for plaque assay, which could contribute to lower detection efficiency. Out of six independent cheese-making experiments, infectious H1N1 was detected only in the whey from 1 experiment (cream cheese). This could be partially explained by the low pH of the whey produced in our lab (4.7 and 5.0), which is unfavorable to H1N1 stability, whereas the pH of the whey in the Nooruzzaman et al. study was 6.6 and 5.8. Also, we do not know the limit of detection (LOD) of the infectivity assay for H1N1 in the whey produced from the cheeses that we made in this study. It is possible that infectious H1N1 particles were present in the whey, but their numbers were below the LOD of the assay; therefore, we could not detect them. We did, however, detect MNV in the curd and whey from both feta-style cheese and cream cheese in all experiments. Nevertheless, the number of detected infectious MNV in the whey from cream cheese is almost 10 times higher than the whey from feta, suggesting some virucidal effect from the whey produced from feta cheese. Another difference between our study and that of Nooruzzaman and colleagues’ is that we used rennet and cultures for making cheeses, which could impact the pH and the microbiome composition of the resultant cheeses.

One of the limitations of this study is employing H1N1 as a surrogate for A(H5N1) viruses, and this is due to the lack of BSL-3 facility for handling HPAI H5N1. It is also noteworthy that there are differences in the biophysical properties between different strains of A(H5N1) viruses (19), and future studies are required to provide additional values by utilizing the circulating strains of A(H5N) in dairy cattle. Herein, in order to remove the trace amounts of potential inhibitors, including the protease inhibitors, carried over from the samples, the cells were rinsed after removing the inoculum. Furthermore, the overlay was subsequently supplemented with trypsin to ensure the proper replication of the virus for plaque assay, therefore more accurately representing the amount of infectious viral particles present in the cheese/whey. Nevertheless, we observed only a limited reduction in the number of infectious viral particles during the cheese-making process.

We next examined the stability of H1N1 in four different types of commercially available unpasteurized milk cheeses and observed that H1N1 is stable in firm cheeses with a gradual decline in infectivity over an 8 week period. This is despite the presence of mold on these cheeses past week 6. The decline in infectivity of H1N1 was more rapid on soft cheese, which could be due to lower pH (4.9) compared to other commercial cheeses tested in this study (pH range 5.4–5.5). The level of infectious H1N1 fell below the LOD after 2 weeks of incubation on soft cheese, which could be due to the fact that the soft cheese samples appeared spoiled, and the virus could have been inactivated by the spoilage flora and their byproducts, or the recovery might have been hindered due to changes in the consistency and composition of the cheese. Our observation is consistent with the recent report on the stability of H5N1 on cheddar cheese during the aging process for 60 days (20). The *D*-value for H1N1 on cheddar was 20.5 days, and in the Nooruzzaman and colleagues’ report, the *D*-value for H5N1 on cheddar was estimated to be 25.5, which is not a drastic difference (20). Another limitation of our cheese aging study is the artificial inoculation, which does not completely represent natural contamination. In artificial inoculation, the virus is not completely protected with fat globules present in the matrix and thus is more exposed to air and prone to drying than the virus that is naturally occurring in milk and then is subjected to cheese making. This could be another reason that we observed a slightly more rapid decline in viral infectivity in cheddar compared to Nooruzzaman and colleagues' study, which used naturally contaminated cheddar cheese. Furthermore, the viral extraction efficiency from the cheese samples could be different between natural contamination and artificial inoculation. Nevertheless, both studies demonstrate that influenza viruses are stable in firm cheeses and that the aging process, without any thermal treatment, might not be sufficient in eliminating the exposure risk.

Furthermore, the soft cream cheese has the lowest fat and the highest moisture content compared to the other studied cheeses, and it has been demonstrated that fat content directly impacts H5N1 survival in dairy products (19), while higher water content allows proliferation of a large variety of microorganisms, leading to the spoilage of cheese (21). We have also observed that high moisture content negatively impacts viral survival.

In this work, we did not analyze the stability of viral RNA, as it has been shown by us and others that detection of viral RNA for both influenza viruses and caliciviruses does not necessarily correspond to viral infectivity (15, 22, 23). Therefore, we expect that the viral RNA levels remain unchanged during the 8 week period, consistent with the observation made by Nooruzzaman and colleagues (17).

In summary, our study implies that the cheese-making process from raw milk might not be sufficient for complete inactivation of H5N1 in contaminated cheeses. However, our recent work has demonstrated that thermization of milk prior to cheese making could be efficient in reducing and even eliminating the risk of exposure to H5N1 through consumption (24). It is notable that there are remaining knowledge gaps regarding the risk of human infection with H5N1 through ingestion. A few H5N1 human illnesses have been reported with unexplained sources, and recently, the U.S. Centers for Disease Control and Prevention outlined the risks of H5N1 in unpasteurized dairy products (25), while infection of cats and mice through ingestion of contaminated H5N1 raw milk has been reported (3, 4). It is still unclear whether exposure through consumption of dairy products contaminated with the current strains of HPAI viruses would result in infection in humans. Given that H5N1 infection among livestock is frequent in many regions of the United States, continuous research, surveillance, and risk assessment of circulating HPAI viruses in livestock, meat, and dairy products could help in understanding and mitigating the impact of HPAI on public health and the agrifood sector.
